# Psychological empowerment and challenge-oriented organizational citizenship behavior: a dual process model

**DOI:** 10.3389/fpsyg.2024.1432260

**Published:** 2024-12-12

**Authors:** Qingquan Xu, Shan Liu, Haishen Huang

**Affiliations:** ^1^Guangdong Huagong Jingzhuo Enterprise Management Consulting Co., Ltd., Guangzhou, China; ^2^School of Business Administration, South China University of Technology, Guangzhou, China

**Keywords:** psychological empowerment, social exchange theory, challenge-oriented organizational citizenship behavior, perceived insider status, affective commitment

## Abstract

**Introduction:**

Encouraging proactive behavior among employees is a crucial pathway for companies to adapt swiftly and gain a competitive edge. In contrast to other forms of organizational citizenship behavior that tend to preserve existing work practices within the organization, Challenge-Oriented Organizational Citizenship Behavior (COCB) aims to improve upon these by offering constructive suggestions and altering employees’ work methods, policies, and procedures for the benefit of the organization. However, not all employees are willing to engage in COCB, even when the organization actively encourages participation. Departing from traditional perspectives on workplace environments and leadership, we propose that psychological empowerment at the individual level may be a precursor to employees’ engagement in COCB.

**Methods:**

Based on social exchange theory, this study examines how employees’ psychological empowerment affects their challengeoriented organizational citizenship behavior (COCB), including the mediating roles of perceived insider status and affective commitment, as well as the moderating role of Chinese traditionality. Data was collected from 223 employees of manufacturing, IT, finance and service enterprises in Southern China using a multi-source and multi-wave survey.

**Results:**

Results of the Structural Equation Modeling (SEM) show that psychological empowerment can positively affect COCB. Both perceived insider status and affective commitment act as mediators in the relationship between psychological empowerment and COCB. Moreover, Chinese traditionality has a moderating effect on the relationship between psychological empowerment and these mediators, and also the indirect effect of psychological empowerment on COCB. Specifically, the indirect effect was stronger when employees with lower levels of Chinese traditionality.

**Discussion:**

This study provides a theoretical foundation for the individual-level antecedents of COCB, elucidating how the social exchange process between employees and the organization is transformed into COCB through psychological empowerment.

## 1 Introduction

After COVID-19 subsides, companies are under intense pressure to adapt to a more complex and volatile business environment due to slowing global economic growth and rapid technological advances. In this context, encouraging proactive behavior among employees is an important way for companies to adapt quickly to gain competitive advantages (Zampetakis and Arvanitis, 2024). Challenge-oriented organizational citizenship behavior (COCB) is a typical proactive behavior, which is defined as employees’ voluntary participation in constructive actions to benefit the organization by making constructive suggestions and changing their work methods, policies, and procedures (Chen et al., 2020; MacKenzie et al., 2011). Allam et al. (2021) explain that workplace spirituality involves individuals seeking meaning in their work environment, forming deep connections with others, and integrating personal values with their work experience. However, this study does not consider citizenship as a component of workplace spirituality. In contrast with other forms of organizational citizenship behavior that tend to maintain the existing work practices within organizations, COCB aims to improve them (De Clercq et al., 2024). Therefore, COCB is crucial for the survival and development of organizations because it can improve organizational efficiency and effectiveness (Chiaburu et al., 2017; MacKenzie et al., 2011).

Despite its importance, however, not all employees exhibit COCB, even when organizations actively encourage participation. This raises critical questions: What motivates some employees to take initiative while others remain disengaged? How can organizations create conditions that foster greater levels of COCB across the workforce? Existing literature has identified workplace characteristics (e.g., service innovation culture, Baradarani and Kilic, 2018) and leader-related factors (e.g., inclusive leadership, Chen et al., 2020) as critical antecedents of COCB. Besides, one factor that is increasingly recognized as a potential driver of COCB is psychological empowerment, which is defined as “a subjective, cognitive, and attitudinal process that helps individuals feel effective, competent and authorized to carry out a task” (Llorente-Alonso et al., 2024). Employees who feel empowered are more likely to challenge the status quo and pursue initiatives that can benefit the organization (Lin et al., 2023). However, the pathways through which psychological empowerment influences CO-OCB remain underexplored, necessitating further investigation.

In terms of research on COCB, there have been studies exploring its antecedents and driving mechanisms from various perspectives, including cognition-based perspective (Younas et al., 2021), conservation of resources (Chen et al., 2020), organizational identity (Seppälä et al., 2012), and stress (Li and Wang, 2022). However, according to social exchange theory, the exchange between employees and organizations is multifaceted, including both economic and socioemotional aspects (Kim, 2014; Xu et al., 2022). The exchange quality can significantly affect the COCB of employees. First, high-quality reciprocal relationships tend to enhance employees’ trust in their organization, fostering their positive attitudes and behaviors in return. Positive affective reactions are often a manifestation of the high-quality social exchange in the employee-organization relationship (Zagenczyk et al., 2021), so employees’ psychological empowerment may not only foster their positive perceptions and attitudes toward the organization but also induce their positive affective states (Llorente-Alonso et al., 2024).

Accordingly, this study introduces perceived insider status and affective commitment to explore their mediating roles in the relationship between psychological empowerment and employees’ COCB from the perspective of reciprocal exchange. Notably, social exchange theory also posits that the exchange process can be influenced by individual factors like personality traits, values, and cultural background (Cropanzano et al., 2017; Kumar et al., 2024). Therefore, it is necessary to consider Chinese cultural and traditional factors when studying social exchange in the Chinese context (Tsui A. S., 2007; Lin et al., 2018). Therefore, this research incorporates Chinese traditionality as a moderator. Since traditionality provides individuals with frameworks for interpreting and evaluating specific management practices, it may influence the social exchange processes related to empowerment that affect individuals’ cognition and emotions (Farh et al., 1997). It may also buffer the effect of psychological empowerment, indirectly affecting COCB through perceived insider status and affective commitment. Thus, the present study additionally investigates whether Chinese traditionality plays a moderating role in the indirect effects of psychological empowerment on COCB through cognitive and affective processes.

Overall, this study aims to investigate the impact of psychological empowerment on employees’ COCB in the Chinese context. It focuses on exploring the mediating roles of perceived insider status and affective commitment as well as the moderating role of Chinese traditionality from the perspective of social exchange theory. By doing so, this study responds to the call for considering the Chinese cultural context in organizational management research (Tsui A. S., 2007; Jia et al., 2012). Furthermore, by clarifying the mechanisms through which psychological empowerment influences employee COCB, this study contributes to expanding the research on psychological empowerment and OCB. In practice, this study offers valuable insights for managing COCB within the Chinese cultural context, thereby fostering organizational innovation and change in Chinese enterprises.

## 2 Theoretical framework and hypotheses

### 2.1 Social exchange theory

Social exchange theory suggests that individuals must adhere to a certain exchange principle—the reciprocity principle—in order to sustain a mutually beneficial relationship during social interaction (Ahmad et al., 2023). This principle requires the recipient to act in a way advantageous to the other person to foster a cycle of reciprocation that motivates social exchange behavior. The rewards and benefits gained from such interactions enhance identification and attachment among the participants, increasing their enthusiasm to sustain the relationship (Cropanzano and Mitchell, 2005). In addition, individual characteristics will affect these social exchange relationships (Cropanzano et al., 2017).

Social exchange theory offers a crucial theoretical foundation for this study because the relationship between an organization and its employees is fundamentally a social exchange (Meira and Hancer, 2021; Ahmad et al., 2023; Xu et al., 2022). Both parties are expected to engage in behaviors that benefit the other. Psychological empowerment implies that employees perceive the benefits and values provided by the organization as the exchanger (Llorente-Alonso et al., 2024), which in turn fosters their identification with and attachment to the organization (affective commitment) as the beneficiaries. Simultaneously, the benefits provided by the organization serve as a crucial criterion for employees to assess their status as “insiders” (perceived insider status; Stamper and Masterson, 2002). To preserve this beneficial relationship, employees must act in ways that favor the organization, motivating them to engage in COCB crucial for the organization’s success (Lin et al., 2023). Employees with different levels of traditionality may perceive social exchange relationships differently, which in turn affects their return to the organization.

### 2.2 Psychological empowerment, perceived insider status and COCB

Empowerment is commonly understood as a management practice that facilitates the distribution of power, primarily through superiors delegating authority to subordinates and assigning tasks, known as structural empowerment (Monje-Amor et al., 2021; Lu et al., 2024). Traditional perspectives on empowerment have predominantly focused on the act of delegating power, overlooking the psychological experiences of those being empowered. However, research indicates that structural empowerment cannot substitute for the actual perception of empowerment by employees, and the empowerment recognized and internalized by employees can play a greater role (Monje-Amor et al., 2021). This insight has led to the emergence of the concept of psychological empowerment, which is increasingly drawing academic attention. Psychological empowerment refers to an individual’s internalization or psychological interpretation of structural empowerment, offering a more precise reflection of an employee’s sense of authorization within an organization (Llorente-Alonso et al., 2024). It is important to recognize that structural empowerment is an essential requirement for psychological empowerment (Singh and Sarkar, 2019; Monje-Amor et al., 2021). Psychological empowerment can have a positive impact on employee work attitudes (Shah et al., 2019; Abbasi et al., 2021), behavioral performance (Chiang and Hsieh, 2012; Travis Maynard et al., 2014; Javed et al., 2017; Wen et al., 2023), and even organizational performance (Iqbal et al., 2020; Pacheco and Coello-Montecel, 2023).

Social exchange theory posits that in order to maintain a social exchange relationship between employees and organizations, the principle of reciprocity must be followed (Ahmad et al., 2023). Psychological empowerment is an employee’s subjective evaluation of leader delegation and resource sharing. A high level of psychological empowerment means that employees perceive more support and trust from the organization, making them more willing to take on work beyond their scope of responsibility to repay the organization (Llorente-Alonso et al., 2024; Wen et al., 2023; Pacheco and Coello-Montecel, 2023), such as COCB. COCB is a form of extra-role behavior, which represents a voluntary, transformative behavior by employees aimed at organizational growth (Choi, 2007; Seppälä et al., 2012; Li et al., 2024). Furthermore, social exchange theory suggests that risk assessment is the starting point of people’s social relationships and that the results of this assessment will affect the way people perceive and act in exchange relationships (Molm et al., 2000; Clark, 2016). Employees with higher psychological empowerment tend to have a higher sense of self-efficacy (Fong and Snape, 2015; Huang, 2017), which on the one hand reduces their perceived risks associated with COCB. On the other hand, their increased willingness to take risks also makes them more likely to exhibit COCB. Therefore, this study proposes that:

*Hypothesis* 1: Psychological empowerment is positively related to employees’ COCB.

Based on the theory of social exchange, the relationship between an organization and its employees is fundamentally a kind of social exchange (Meira and Hancer, 2021; Ahmad et al., 2023). Authorization can improve people’s exploratory and creative behavior (Brändle et al., 2023), because empowerment makes people have more control over their own thoughts and behaviors. Granting psychological autonomy enables employees to recognize the rewards and incentives provided by the organization (Llorente-Alonso et al., 2024). The value of these rewards and incentives often helps employees distinguish between being “insiders” or “outsiders” within the organization, thereby affecting employees’ perceived insider status. Employees who are more psychologically empowered may perceive that they have gained more power and resources from the organization. This perception fosters their trust and recognition of the organization (Hill et al., 2014; Horng et al., 2016), leading them to identify themselves as insiders with a favorable exchange relationship with the organization. Conversely, employees who perceive a lack of psychological empowerment are less likely to recognize organization due to the belief that the organization has allocated them fewer resources, and they may perceive themselves as “outsiders” thus diminishing their enthusiasm to contribute to the organization (Llorente-Alonso et al., 2024). Based on this, we put forward the following assumptions:

*Hypothesis* 2: Psychological empowerment is positively related to perceived insider status.

As an employee’s sense of belonging within the organization, perceived insider status plays a crucial role in shaping the relationship between the organization and its employees (Wang and Kim, 2013; Ademolu, 2024). Employees will evaluate their “insides” (Perceived Insider Status) according to the resources provided by the organization (Stamper and Masterson, 2002). Generally, organizations offer different incentives and rewards to those they consider “insiders” compared to “outsiders,” influencing employees’ perceptions and, consequently, their work attitudes and behaviors (Chen and Aryee, 2007; Kim et al., 2019). Employees will make efforts to maintain this beneficial relationship, thus participating in this kind of COCB beneficial to the organization (Lin et al., 2023). Thus, they are more willing to return to the organization through more extra-role behaviors (Wang and Kim, 2013; Hui et al., 2015; Li et al., 2024), potentially including more COCB. Therefore, we propose the following hypothesis:

*Hypothesis* 3: Perceived insider status is positively related to COCB.

Social exchange theory (SET) emphasizes reciprocity and resource sharing, providing a framework for understanding interactions between individuals and entities (Gong and Yi, 2021; Ahmad et al., 2023). Based on SET, we propose that when employees experience psychological empowerment, they are more likely to engage in COCB (citizenship-oriented organizational citizenship behaviors) by enhancing their perceived insider status. Psychological empowerment influences employees’ attitudes toward the organization, subsequently shaping their cognitions and behaviors (Shah et al., 2019; Abbasi et al., 2021; Wen et al., 2023). When employees feel a higher sense of psychological empowerment, they are more inclined to recognize their value and status within the organization (Llorente-Alonso et al., 2024), which elevates their perceived insider status and, in turn, makes them more likely to engage in COCB. Based on the above analysis, we propose the following hypotheses:

*Hypothesis* 4: The relationship between psychological empowerment and COCB is mediated by perceived insider status.

### 2.3 Psychological empowerment, affective commitment and COCB

According to social exchange theory, the quality of the relationship between exchange parties affects the process of exchange, and affective commitment is an important indicator of the relationship quality between an organization and its employees (Judge and Kammeyer-Mueller, 2012; Mazzei et al., 2023). Psychological empowerment reflects the resources provided to employees at the organizational level (Llorente-Alonso et al., 2024), which enhances the quality of the relationship between employees and the organization. When employees feel that the organization or their leaders are providing them with increased authority and resources, such organizational support will enhance their gratitude and trust (Gigliotti et al., 2019), which in turn will facilitate their psychological attachment to and affective identification with the organization (Rhoades et al., 2001; Fong and Snape, 2015). Accordingly, we argue that employees’ psychological attachment fosters feelings of gratitude and trust, which, in turn, enhance their affective commitment to the organization. Based on this, we propose the following hypothesis:

*Hypothesis* 5: Psychological empowerment is positively related to affective commitment.

Employee affective commitment to the organization is often characterized by strong emotional bonds, identification, and loyalty (Allen et al., 2011; Kim et al., 2023). Affective commitment strengthens employees’ connection with the company, impacting the organization’s reputation and aiding new employees in adapting to the workplace (Kim et al., 2023; Mazzei et al., 2023). Employees with high affective commitment are more inclined to identify with the organization, possess a stronger sense of collectivism, and are more likely to engage in extra-role behaviors. As a result, employees are more inclined to invest additional time and energy to engage in COCB as a return to the organization (Wang Q. et al., 2014; Guo et al., 2022). Consequently, we hold that the higher the level of employees’ affective commitment, the deeper their cognitive and emotional connection to the organization. This increased connection motivates them to engage in extra-role behaviors, thereby promoting COCB. Therefore, this study proposes that:

*Hypothesis* 6: Affective commitment is positively related to COCB.

Drawing from social exchange theory, we suggest that psychological empowerment, by enhancing organizational resources and support, leads to a stronger affective commitment among employees. This affective commitment then serves as a motivational mechanism, translating the psychological empowerment into increased engagement in COCB. Psychological empowerment positively impacts employees by increasing their work engagement (Llorente-Alonso et al., 2024; Wen et al., 2023), enhancing their affective commitment. Affective commitment, in turn, strengthens organizational identification and improves work performance (Mazzei et al., 2023). We believe that this ultimately promotes employees’ COCB (citizenship-oriented organizational citizenship behaviors). Specifically, the trust and emotional bonds developed through psychological empowerment strengthen employees’ emotional attachment to the organization, which subsequently encourages them to go beyond their formal roles to contribute to COCB. Therefore, we propose the following hypothesis:

*Hypothesis* 7: The relationship between psychological empowerment and COCB is mediated by affective commitment.

### 2.4 The moderating role of Chinese traditionality

Social exchange theory posits that individuals reciprocate the benefits they receive in social interactions to maintain relationships. When treated unfairly, individuals may respond negatively to the exchange (Cropanzano and Mitchell, 2005; Roch et al., 2019; Ahmad et al., 2023). However, reactions to unfair treatment can vary, with cultural values influencing perceptions of exchange relationships (Cropanzano et al., 2017).

Chinese traditionality reflects cognitive attitudes and behavioral patterns in the context of traditional Chinese culture, which encompasses obedience to authority, filial piety, respect for ancestors, and contentment, effectively capturing the characters and values of traditional Chinese individuals (Farh et al., 1997; Zhang et al., 2014; Li et al., 2023). Studies have demonstrated that when employees are empowered to lead, they are more likely to view themselves as an insider, and this relationship can be affected by their traditional cultural values (Chen and Aryee, 2007). Studies also indicate that employees’ cognition (Zhang et al., 2014; Guan et al., 2016), affect (Wang H. et al., 2014; Wang et al., 2020), behavior (Li and Yu, 2017; Hu et al., 2022; Guang et al., 2024), and performance (Liu et al., 2013; Wang and Kim, 2013) vary with their level of Chinese traditionality. Individuals with high traditionality tend to internalize external social norms and traditional morals, meanwhile undervaluing their own worth (Farh et al., 2007). Consequently, employees who have high traditionality may diminish the positive impact of psychological empowerment on their perception of insider status due to their strong adherence to social norms. In contrast, those with a low traditionality tend to view their relationship with the organization or leader as a social exchange (Li and Yu, 2017), with fewer restrictions from social norms and traditions, and they will focus more on their inner experiences. Therefore, for employees with low traditionality, psychological empowerment plays a more significant role in shaping their perceived insider status, strengthening the positive link between psychological empowerment and the perception of insider status. Therefore, this study proposes that:

*Hypothesis* 8a: Chinese traditionality moderates the positive relationship between psychological empowerment and perceived insider status. The positive relationship is stronger for employees with low traditionality than those with high traditionality.

*Hypothesis* 8b: Chinese traditionality moderates the indirect effect of psychological empowerment on COCB through perceived insider status. The indirect effect is stronger for employees with low traditionality than those with high traditionality.

When organization members are influenced by traditional culture, they often believe that their actions should align with authority and social norms (Li et al., 2023). While individual internal experiences influence emotional responses to the organization, these experiences are, to a degree, “yielded” to Chinese traditionality. Under the influence of traditional values such as contentment, dedication, and conscientiousness, employees with high traditionality tend to develop a strong affective identification with the organization (Farh et al., 2007; Tasoulis et al., 2024). This occurs regardless of the level of psychological empowerment they experience, leading to a less pronounced impact of psychological empowerment on their organizational commitment. Conversely, when Chinese traditionality is low, their positive psychological orientation is primarily derived from both personal and organizational influences, rather than conforming to prevailing traditional norms. In such cases, the level of employee psychological empowerment has a more prominent impact on affective commitment. Thus, the higher the psychological empowerment, the more likely they are to feel valued and identify with the organization’s values. This leads to a greater willingness to contribute and sacrifice for the benefit of the organization (Qing et al., 2020), resulting in higher affective commitment. Therefore, this study proposes that:

*Hypothesis* 9a: Chinese traditionality moderates the positive relationship between psychological empowerment and affective commitment. The positive relationship is stronger for employees with low traditionality than those with high traditionality.

*Hypothesis* 9b: Chinese traditionality moderates the indirect effect of psychological empowerment on COCB through affective commitment. The indirect effect is stronger for employees with low traditionality than those with high traditionality.

## 3 Methods

### 3.1 Participants and procedure

To test our hypotheses, we utilized a questionnaire survey to collect sample data. Participants were recruited from different companies in various industries (i.e., manufacturing, IT, finance and service) in South China, thereby enhancing the external validity of our findings. Our reason for choosing these companies is that they often face fierce competition and often require product, technology, or service innovation. Therefore, employees of these companies often need to change the status quo, leading to frequent occurrences of COCB. Adopting snowball sampling method, we contacted managers who held leadership positions in companies through the alumni directory of a famous university in South China and asked them to recommend at least three subordinates to participate in the survey. In this way, we recruited a total of 316 full-time employees who agreed to participate. All questionnaires were sent via WeChat, an instant messaging mobile application with more than 1 billion active users, which is often used for questionnaire surveys in China. Before distributing questionnaires, we informed all participants about the purpose and procedures of the research. To obtain high data quality and high response rates, we promised all the participants that their answers would only be used for academic research and that they would receive a reward of RMB 5 after completing the questionnaire, and emphasized the importance of their honest responses.

To reduce common method variance (Podsakoff et al., 2012), we collected dyadic data from employees and their immediate supervisors at two different time points (Time 1 and Time 2). To be specific, at Time 1, employees were asked to complete their self-assessments of their psychological empowerment, perceived insider status, affective commitment, and traditionality. At Time 2 (1 month later after Time 1), supervisors were asked to evaluate the COCB of their subordinates. In the initial survey, 316 questionnaires were sent, and 292 were returned, resulting in a 92.41% response rate. In the second wave of the survey, 292 questionnaires were distributed, and 255 were returned, with a response rate of 87.33%. Based on recommendations, the sample size for structural equation modeling should not be fewer than 200 (Wolf et al., 2013). After removing invalid data, we finally obtained 223 valid employee-leader paired questionnaires, achieving a response rate of 70.57%.

The demographics of the samples were as follows: 38.10% were male, the mean age of the respondents was 30.56 (*SD* = 6.88), educational attainment was 56.10%, bachelor’s degree holders were 7.60%, postgraduate degree holders and above, 24.20% were college degree holders, and 11.20% were less than college degree holders, the average number of years of work experience of the respondents was 2.95 (*SD* = 1.36), and in terms of positional hierarchy, 68.60% of the respondents were general employees, 21.50% were junior managers, and 8.10% were middle managers.

### 3.2 Measures

This research used scales that have been published and extensively verified for accuracy. The researcher adhered to a rigorous translation and back-translation procedure to guarantee the accuracy of the scales in the Chinese setting. This research utilized a 5-point Likert scale, with 1 indicating “strongly disagree” and 5 indicating “strongly agree.”

Psychological empowerment was measured using the 12-item scale developed by Spreitzer (1995). The scale contains four dimensions (meaning, competency, self-determination, and impact). The sample items for the meaning dimension include “The work I do is meaningful to me.” The sample items for the competency dimension include “I am confident about my ability to do my job.” The sample items for the self-determination dimension include “I can decide on my own how to go about doing my work,” and the sample items for the impact dimension include “My impact on what happens in my department is large” (*α* = 0.82).

The six-item scale from Stamper and Masterson was utilized to assess the perceived insider status (2002). The sample items include “I feel very much a part of my work organization” (α = 0.77).

Affective commitment was measured using the 6-item scale developed by Meyer et al. (1993). The sample items include “This organization has a great deal of personal meaning for me” (α = 0.88).

Chinese traditionality was measured using a 5-item scale developed by Farh et al. (1997). Example items include “Those who are respected by parents should be respected by their children” (α = 0.70).

COCB was measured using a 5-item scale developed by MacKenzie et al. (2011). Example items include “This employee often tries to recommend changes in organizational rules or policies that are nonproductive or counterproductive” (α = 0.71).

This study controlled for the following variables to prevent their interference with the results: gender (1 = male, and 2 = female), age (in years), education (1 = high school, 2 = college, 3 = bachelor, and 4 = master or above), tenure (1 = lower than 2 years, 2 = 2–4 years, 3 = 5–8 years, 4 = more than 8 years) and position level (1 = employee, 2 = first-line manager, 3 = middle-level managers).

### 3.3 Analytical strategy

The variables of this study were first examined for their discriminant validity through confirmatory factor analysis (CFA), followed by an initial exploration of the relationship between the variables through descriptive statistical analysis, and finally, the hypotheses formulated in the study were tested using structural equation modeling (SEM) and further evaluated with a bootstrapping method to examine the hypotheses of this study. The software employed for statistical analysis was SPSS 22.0 and Mplus 7.4.

## 4 Results

### 4.1 Confirmatory factor analysis

The variables in this study were tested for their discriminant validity through the comparison of different measurement models. Table 1 shows that the five-factor model fits the data better than the other models, supporting the discriminant validity of the variables.

**Table 1:** Comparison of measurement models (*N* = 223).

Model	*χ*^2^	*df*	*χ*^2^ /*df*	CFI	TLI	SRMR	RMSEA
Model 1 (hypothesized five-factor model)	739.823	477	1.551	0.924	0.911	0.078	0.050
Model 2 (four-factor model: combines PIS and AC)	774.279	481	1.610	0.915	0.901	0.082	0.052
Model 3 (three-factor model: combines PIS, AC and COCB)	903.341	484	1.866	0.879	0.860	0.085	0.062
Model 4 (three-factor model: combines PE, PIS and AC)	926.678	484	1.915	0.872	0.852	0.084	0.064
Model 5 (two-factor model: combines PE, PIS, AC and COCB)	1042.391	486	2.145	0.840	0.815	0.087	0.072
Model 6 (one-factor model)	1825.865	487	0.749	0.599	0.538	0.099	0.111

PE, psychological empowerment; PIS, perceived insider status; AC, affective commitment; CFI, comparative fit index; TLI, Tucker-Lewis index; SRMR, standardized root mean square residual; RMSEA, root mean square error of approximation.

### 4.2 Descriptive statistics

Table 2 displays the results of the mean, standard deviation, and correlations of the core variables of this study. Results show that there is a strong correlation between psychological empowerment and perceived insider status, affective commitment, and COCB. The findings provided preliminary support for the proposed hypotheses.

**Table 2:** Descriptive and correlational statistics (*N* = 223).

Variable	1	2	3	4	5	6	7	8	9	10
1 Gender	—									
2 Age	−0.194^**^	—								
3 Education	0.018	−0.004	—							
4 Tenure	−0.170^*^	0.816^**^	−0.100	—						
5 Position level	−0.196^**^	0.392^**^	0.111	0.341^**^	—					
6 PE (T1)	−0.142^*^	0.026	−0.029	0.010	0.135^*^	**(0.819)**				
7 PIS (T1)	−0.006	0.002	−0.212^**^	0.078	0.117	0.336^**^	**(0.767)**			
8 AC (T1)	−0.222^**^	0.022	−0.127	0.048	0.119	0.548^**^	0.454^**^	**(0.881)**		
9 CT (T1)	−0.083	0.185^**^	−0.101	0.185^**^	0.102	−0.066	−0.159^*^	−0.071	**(0.703)**	
10 COCB (T2)	−0.287^**^	0.091	−0.173^*^	0.077	0.267^**^	0.396^**^	0.337^**^	0.415^**^	0.039	**(0.709)**
Mean	1.620	30.560	2.610	2.950	1.380	3.609	3.781	3.882	3.251	3.573
Standard deviation	0.487	6.879	0.788	1.364	0.635	0.433	0.595	0.544	0.572	0.535

T1, Time 1; T2, Time 2; PE, psychological empowerment; PIS, perceived insider status; AC, affective commitment; CT, Chinese traditionality. ^**^p < 0.01; ^*^p < 0.05. The bold values represent Cronbach’s α coefficient.

**Figure 1:**
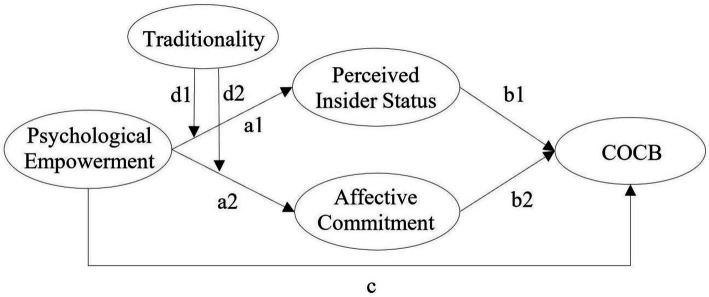
The proposed theoretical model and main paths.

Figure 1 shows the proposed theoretical model and main paths. a1 represents the impact of psychological empowerment on perceived insider status, a2 represents the effect of psychological empowerment on affective commitment, b1 represents the impact of perceived insider status on COCB, b2 represents the impact of affective commitment on COCB, c represents the direct effect of psychological empowerment on COCB, and d1 and d2 represent the moderating effects of Chinese traditionality. Furthermore, a1 × b1 indicates the extent to which perceived insider status mediates, a2 × b2 suggests the extent to which affective commitment mediates, and a1 × b1 + a2 × b2 + c indicates the total effect of psychological empowerment on COCB.

### 4.3 Main and mediating effects tests

Table 3 shows that psychological empowerment has a significant positive influence on COCB (*β* = 0.432, SE = 0.074, *p* < 0.001), which supports Hypothesis 1. Psychological empowerment has a significant positive influence on perceived insider status (*β* = 0.413, SE = 0.086, *p* < 0.001), which supports Hypothesis 2. Perceived insider status has a significant positive influence on COCB (*β* = 0.150, SE = 0.059, *p* < 0.05), which supports Hypothesis 3. The significance of perceived insider status as a mediator between psychological empowerment and COCB is supported (indirect effect = a_1_ × b_1_ = 0.062, SE = 0.028, *p* < 0.05), which supports Hypothesis 4. We used the method developed by Sobel (1982) to test the mediating effect. Psychological empowerment has a significant positive influence on affective commitment (*β* = 0.657, SE = 0.071, *p* < 0.001), which supports Hypothesis 5. Affective commitment has a significant positive influence on COCB (*β* = 0.172, SE = 0.072, *p* < 0.05), which supports Hypothesis 6. The mediating effect of affective commitment between psychological empowerment and COCB is also significant (indirect effect = a_2_ × b_2_ = 0.113, SE = 0.049, *p* < 0.05), which supports Hypothesis 7.

**Table 3:** Results of path analysis.

Path	Coefficients	Standard errors
**a**_**1**_ (psychological empowerment → perceived insider status)	0.413^***^	0.086
**a**_**2**_ (psychological empowerment → affective commitment)	0.657^***^	0.071
**b**_**1**_ (perceived insider status → COCB)	0.150^*^	0.059
**b**_**2**_ (affective commitment → COCB)	0.172^*^	0.072
**c** (direct effect, psychological empowerment → COCB)	0.258^**^	0.085
**d**_**1**_ (the moderating effects 1)	−0.350^*^	0.141
**d**_**2**_ (the moderating effects 2)	−0.264^*^	0.116
**a**_**1**_ **× b**_**1**_ (the mediating effects of perceived insider status)	0.062^*^	0.028
**a**_**2**_ **× b**_**2**_ (the mediating effects of affective commitment)	0.113^*^	0.049
**a**_**1**_ **× b**_**1**_ **+ a**_**2**_ **× b**_**2**_ **+ c** (the total effect)	0.432^***^	0.074

*N* = 223. ^***^p < 0.001; ^**^p < 0.01; ^*^p < 0.05.

We adopted the Bootstrapping method (Shrout and Bolger, 2002) to further examine the mediating effects of perceived insider status and affective commitment (see Table 4). After 5,000 Bootstrapping re-sampling, the 95% confidence interval for the mediating effect of perceived insider status was [0.016, 0.130], which excluded 0, indicating that the mediating effect of perceived insider status was significant, Hypothesis 4 is further supported. While the 95% confidence interval for the mediating effect of affective commitment was [0.031, 0.226], which excluded 0, indicating that the mediating effect of affective commitment was significant, and Hypothesis 7 is further supported.

**Table 4:** Bootstrapping results for the mediation effects.

Path	Estimate	95% CI	Percentage of explanation (%)
1. Indirect Path 1 (Psychological Empowerment →Perceived Insider Status → COCB)	0.062^*^	[0.016, 0.130]	14.352
2. Indirect Path 2 (Psychological Empowerment → Affective Commitment → COCB)	0.113^*^	[0.031,0.226]	26.157
3. Direct Path (Psychological Empowerment → COCB)	0.257^*^	[0.093, 0.407]	59.491
4. Full path	0.432^***^	[0.306, 0.575]	—

*N* = 223, Bootstrap = 5,000. CI, confidence interval, ^***^p < 0.001; ^**^p < 0.01; ^*^p < 0.05.

### 4.4 Moderating effects test

As shown in Table 3, the interaction term of psychological empowerment and Chinese traditionality has a significantly negative effect on perceived insider status (*β* = −0.350, SE = 0.141, *p* < 0.05). This implies that Chinese traditionality has a moderating effect on the relationship between psychological empowerment and perceived insider status, and Hypothesis 8a was supported. Additionally, the interaction term of psychological empowerment and Chinese traditionality has a significantly negative effect on affective commitment (*β* = −0.264, SE = 0.116, *p* < 0.05), suggesting that Chinese traditionality has a moderating effect on the relationship between psychological empowerment and affective commitment, and Hypothesis 9a was also supported.

**Figure 2:**
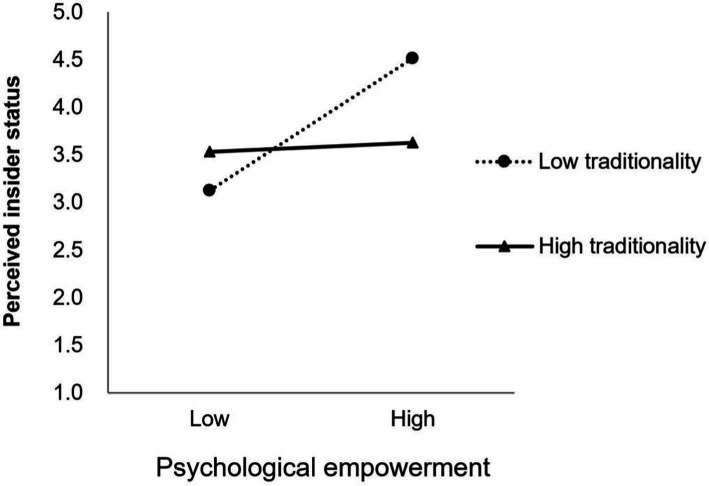
The moderating effect of traditionality on the relationship between psychological empowerment and perceived insider status.

**Figure 3:**
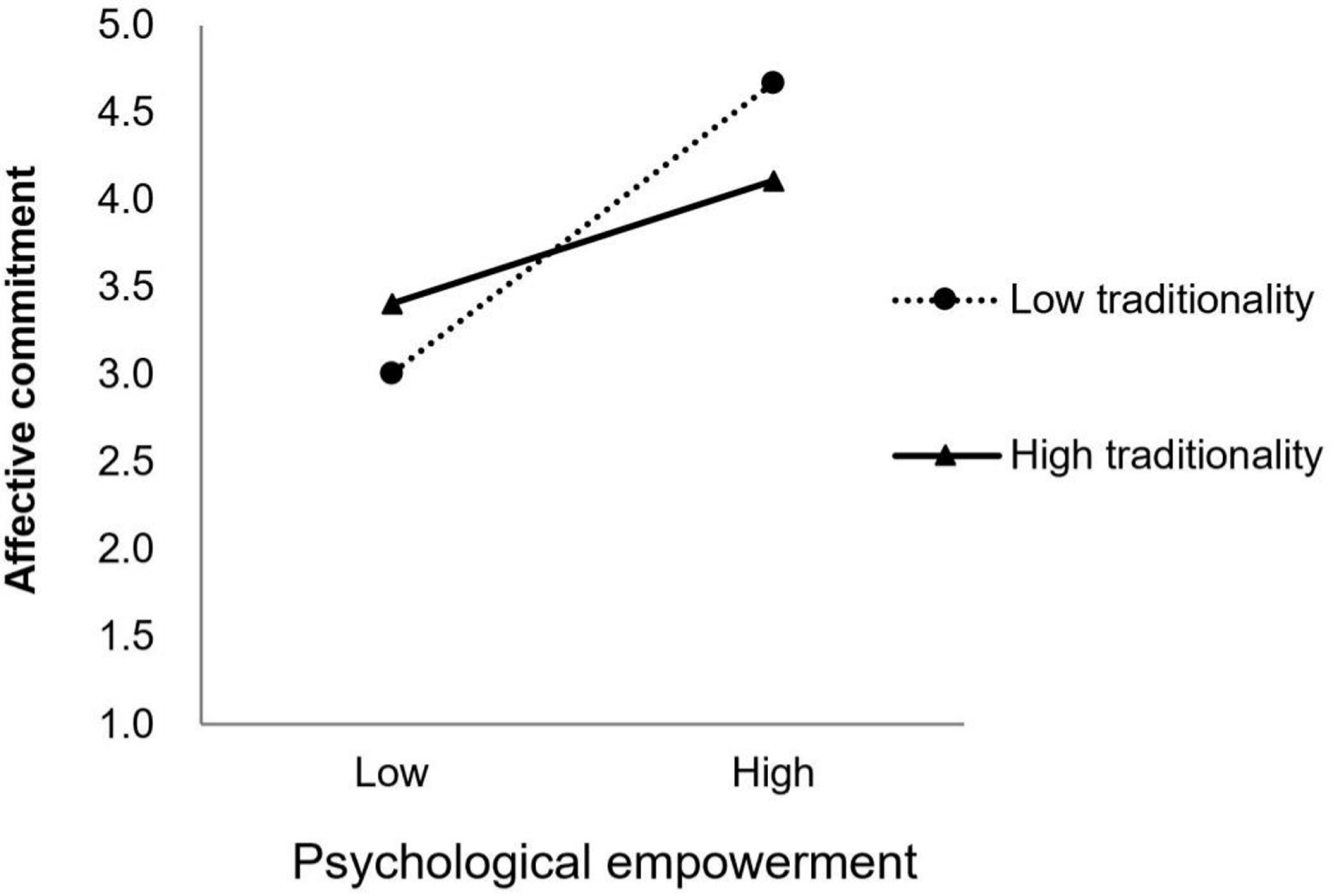
The moderating effect of traditionality on the relationship between psychological empowerment and affective commitment.

Additionally, the simple slope test in Figure 2 indicated that psychological empowerment has a more pronounced effect on the perceived insider status for employees with lower traditionality (*β* = 0.092, *p* < 0.05), whereas for those with higher traditionality, the effect of psychological empowerment on perceived insider status is not significant (*β* = 0.032, *p* > 0.05), and Hypothesis 8a was further supported. As can be seen in Figure 3, for employees with lower traditionality, the positive relationship between psychological empowerment and affective commitment is significant (*β* = 0.139, *p* < 0.05), and the positive relationship between psychological empowerment and affective commitment is also significant for those with higher traditionality (*β* = 0.087, *p* < 0.05), but the effect is weaker. Thus, Hypothesis 9a was also further supported.

This study employed Bootstrapping to examine the moderated mediation effects. When Chinese traditionality is high, the 95% confidence interval of the indirect effect of psychological empowerment on COCB via perceived insider status is [−0.002, 0.106], which contains 0, indicating that the indirect effect is not significant; when Chinese traditionality is low, the 95% confidence interval of the indirect effect of psychological empowerment on COCB through perceived insider status is [0.025, 0.177]. The absence of a 0 in this interval implies that the indirect effect is valid. The 95% confidence interval for the difference between the high and low subgroups is [−0.139, −0.013], which does not include 0. Additionally, the 95% confidence interval of the indirect effect of psychological empowerment on COCB via affective commitment when the traditionality is high is [0.023, 0.183]. When the traditionality is low, the 95% confidence interval is [0.038, 0.275]. The absence of a 0 in these intervals implies that these indirect effects are valid. The 95% confidence interval for the difference between the high and low subgroups is [−0.134, −0.011], indicating no presence of 0. Thus, the moderated mediation effects of the dual paths are both significant, Hypotheses 8b and 9b were supported.

## 5 Discussion

In this study, we analyzed the direct effect of psychological empowerment on customer-oriented citizenship behavior (COCB). Additionally, we examined the mediating roles of perceived insider status and affective commitment in the relationship between psychological empowerment and COCB, as well as the moderating role of Chinese traditionality. Grounded in social exchange theory, we hypothesized that psychological empowerment would significantly influence COCB through perceived insider status and affective commitment. Furthermore, we proposed that the effects of psychological empowerment on perceived insider status and affective commitment would be stronger when Chinese traditionality is lower.

The results demonstrated that psychological empowerment positively influenced perceived insider status (*β* = 0.413, SE = 0.086, *p* < 0.001) and affective commitment (*β* = 0.657, SE = 0.071, *p* < 0.001). These findings are consistent with prior research on psychological empowerment (Llorente-Alonso et al., 2024; Lin et al., 2023), indicating that psychological empowerment enhances employees’ positive emotions toward the organization. Perceived insider status and affective commitment also positively impacted COCB (*β* = 0.150, SE = 0.059, *p* < 0.05; *β* = 0.172, SE = 0.072, *p* < 0.05), supporting previous studies (Wang and Kim, 2013; Ademolu, 2024; Allen et al., 2011; Kim et al., 2023) that highlight their crucial roles in fostering positive employee attitudes and behaviors. Furthermore, the mediating effects of perceived insider status and affective commitment between psychological empowerment and COCB were significant (a1 × b1 = 0.062, SE = 0.028, *p* < 0.05; a2 × b2 = 0.113, SE = 0.049, *p* < 0.05). The interaction term between psychological empowerment and Chinese traditionality significantly influenced perceived insider status (*β* = −0.350, SE = 0.141, *p* < 0.05) and affective commitment (*β* = −0.264, SE = 0.116, *p* < 0.05), indicating a significant moderating effect. Additionally, the moderated mediation effects were also significant, supporting all our proposed hypotheses.

### 5.1 Theoretical contributions

First, this study provides theoretical support for the influence of psychological empowerment on constructive organizational citizenship behavior (COCB). Unlike organizational citizenship behavior (OCB), COCB is specifically aimed at improving existing work practices within the organization (De Clercq et al., 2024). Our study addresses the question of why employees engage in COCB. Although prior research has highlighted workplace factors and leadership as significant antecedents of COCB (Baradarani and Kilic, 2018; Chen et al., 2020), it is essential to recognize that COCB is fundamentally an individual-level behavior. Thus, our research offers insights into the personal antecedents of COCB. Drawing on social exchange theory, we demonstrate how psychological empowerment facilitates individual engagement in COCB through social exchange processes. This finding aligns with previous perspectives suggesting that psychological empowerment fosters positive emotional and attitudinal responses among employees toward the organization, thereby eliciting proactive organizational behaviors (Shah et al., 2019; Abbasi et al., 2021; Llorente-Alonso et al., 2024). Specifically, our research highlights how the social exchange process between employees and the organization enhances perceived insider status and affective commitment, subsequently promoting COCB.

Second, this study sheds light on the influence of regional culture on employee behavior and identifies boundary conditions for the impact of psychological empowerment on COCB. We incorporate Chinese traditionality, a cultural element rooted in the Chinese context, into the examination of how psychological empowerment influences COCB. Social exchange processes can be affected by cultural backgrounds (Cropanzano et al., 2017; Li et al., 2023), and it is crucial to consider traditional cultural elements in the study of social relationships within the Chinese cultural setting (Tsui A. S., 2007; Lin et al., 2018). Our findings indicate that employees are more likely to engage in COCB when their level of Chinese traditionality is low. This result is consistent with prior studies, which have shown that individuals with high traditionality tend to internalize external social norms and moral standards, undervalue their own contributions, and exhibit strong emotional attachment to the organization (Farh et al., 2007; Tasoulis et al., 2024). These findings suggest that Chinese traditionality moderates the impact of psychological empowerment on cognitive and emotional behaviors. This underscores the necessity of adapting Western management theories to the unique Chinese cultural environment, addressing the call for context-specific research in the study of organizational management in China (Tsui A. B. M., 2007; Jia et al., 2012). Furthermore, the insights gained from this study enrich the research on psychological empowerment and OCB, offering more tailored guidelines for managing OCB within the Chinese context.

Third, this study contributes to the body of social exchange theory by explicating the social exchange process between psychological empowerment and COCB, with a particular focus on Chinese traditionality as a boundary condition. Social exchange between employees and the organization is multifaceted, encompassing both tangible and emotional aspects (Kim, 2014; Xu et al., 2022). Psychological empowerment, as a significant socio-emotional resource, influences the social exchange process by enhancing perceived insider status and affective commitment, thus affecting employees’ engagement in COCB. This extension of social exchange theory also acknowledges the potential influence of cultural context on social exchange processes (Cropanzano et al., 2017). By illustrating how Chinese traditionality moderates the relationship between psychological empowerment and COCB, our study demonstrates the cultural dimension’s impact on social exchange dynamics. In sum, our research integrates individual characteristics and cultural factors, providing a comprehensive understanding of the social exchange process.

### 5.2 Practical implications

First, it is crucial for companies to not only focus on leader empowering behaviors but also ensure that employees genuinely feel empowered by the organization. This perception helps employees feel the importance the organization places on them, thereby enhancing their positive views of job meaning, competence, autonomy, and impact (Llorente-Alonso et al., 2024). This approach helps to stimulate employees’ abilities and potential, encouraging them to contribute to the organization and take risks. For example, Google is renowned for emphasizing employee autonomy and fostering a culture of innovation. The company integrates psychological empowerment into its management practices in multiple ways. One well-known initiative is Google’s “20% time” policy, which encourages employees to dedicate 20% of their work hours to pursuing innovative projects unrelated to their primary responsibilities. This empowerment strategy enhances employees’ sense of ownership and intrinsic motivation, leading to groundbreaking innovations such as Google Maps and Gmail. Organizations should adopt measures to increase psychological empowerment among employees, thereby promoting career development and job satisfaction while boosting their creativity and sense of belonging.

Second, companies can motivate employees to offer transformative suggestions or engage in innovative behaviors that benefit the organization. This goes beyond creating an empowering atmosphere, it also involves fostering employees’ sense of belonging and strengthening their “insider” beliefs and affective commitment to the organization. Such strategies can enhance their intrinsic motivation to contribute to the organization. This is also an important way for companies to gain a competitive advantage (Zampetakis and Arvanitis, 2024). Toyota, on the other hand, actively encourages all employees to engage in the continuous improvement of production processes. Employees are not only empowered to suggest enhancements but also given the authority to implement these ideas. This approach generates tens of thousands of innovation proposals each year, with a large portion put into action. By involving employees in optimizing processes, Toyota has significantly improved productivity, product quality, and resource efficiency. Besides, the adoption of different leadership or management styles tailored to individual employees is important. For employees with low Chinese traditionality, managers should consider offering them more empowerment and autonomy, while for employees with high traditionality, who tend to have a greater respect for authority and are more inclined to obedience, managers should focus on direction through instructions or guidance. This personalized approach ensures that management styles align effectively with employees’ cultural and personal orientations.

### 5.3 Limitations and future directions

This study has several limitations. First, regarding research design, although it employed a multi-wave and multi-source data collection method, it is still not a strict longitudinal study, and common method bias might still exist to some extent (Podsakoff et al., 2012). Although our study is based on multi-source data (employees and their leaders), conducting psychological experiments to examine the impact of psychological empowerment on employee behavior could significantly enhance the reliability of the research outcomes. Furthermore, since emotions and attitudes are dynamic human states that evolve over time, we suggest that future research on psychological empowerment, perceived insider status, and affective commitment employ experience sampling methods to capture the emotional changes in employees over time (Fisher and To, 2012).

Second, the participants of this study were primarily from enterprises in South China.We explored the moderating effect of Chinese traditionality, which is an important feature and contribution of this study, but it also has limitations. The cultural characteristics from China may limit the generalizability of this research, as it might not apply to Western contexts like the U.S. and the U.K. Organizational culture may affect employee behavior (Watanabe et al., 2024), emphasizing collectivism, Chinese culture may unintentionally strengthen employees’ organizational identification and positive psychological states (Pierce et al., 2018), or lead employees to exhibit higher levels of perceived insider status and affective commitment than actual. Therefore, we suggest future research consider the impact of cultural background on employee psychology and behavior to draw more universally applicable conclusions.

We also recommend future studies explore other antecedents of COCB. While this research provides a theoretical basis for the influence of psychological empowerment on COCB through perceived insider status and affective commitment, we believe team-level factors should be considered. Workplace factors and leadership are important antecedents of COCB (Baradarani and Kilic, 2018; Chen et al., 2020), task-related factors will affect performance (Saleem et al., 2023; Zheng et al., 2022), which may be an important antecedent of COCB. Team-level elements, such as team goals and atmosphere, could be critical to employees’ engagement in COCB. Besides, Workplace environmental factors, such as gossip, can affect employee engagement (Shan et al., 2024), employees who are influenced by gossip may exhibit varying levels of COCB. Investigating these factors can further enrich the research on COCB.

## Data Availability

The raw data supporting the conclusions of this article will be made available by the authors, without undue reservation.
